# Effectiveness of Body Armor Against Shock Waves: Preventing Blast Injury in a Confined Space

**DOI:** 10.7759/cureus.57568

**Published:** 2024-04-03

**Authors:** Nobuaki Kiriu, Daizoh Saitoh, Yasumasa Sekine, Koji Yamamura, Masanori Fujita, Toshiharu Mizukaki, Satoshi Tomura, Tetsuro Kiyozumi

**Affiliations:** 1 Division of Traumatology, Research Institute, National Defense Medical College, Saitama, JPN; 2 Department of Traumatology and Critical Care Medicine, National Defense Medical College, Saitama, JPN; 3 Graduate School of Emergency Medical System, Kokushikan University, Tokyo, JPN; 4 Department of Oral Surgery, National Defense Medical College, Saitama, JPN; 5 Division of Environmental Medicine, Research Institute, National Defense Medical College, Saitama, JPN; 6 Department of Aeronautics and Astronautics, School of Engineering, Tokai University, Kanagawa, JPN

**Keywords:** war, shock wave, confined space, body armor, blast injury

## Abstract

Introduction

Blast injuries in modern society often occur owing to terrorist attacks in confined spaces, particularly in urban settings, indoors, and in vehicles, leading to significant damage. Therefore, it is important to focus on blast injuries in confined spaces rather than in conventional open-field experiments.

Materials and methods

We used an air-driven shock wave generator (blast tube) established indoors in 2017 and conducted basic research to potentially save the lives of patients with blast injuries. Under general anesthesia, pigs were divided into with body armor (BA) and without BA groups. The pigs were fixed in the measurement chamber with their dorsal chest directly exposed to the shock wave. The driving pressure was set at 3.0 MPa to achieve a mortality rate of approximately 50%. A generated shock wave was directly applied to the pigs. Comparisons were made between the groups with respect to cardiac arrest and survival, as well as apnea, bradycardia, and hypotension, which are the triad of blast lung. Autopsies were performed to confirm the extent of the organ damage. Statistical analysis was performed using Fisher's exact test, and statistical significance was set at *p*<0.05. The animal experimentation was conducted according to the protocol reviewed and approved by the Animal Ethics Committee of the National Defense Medical College Hospital (approval number 19041).

Results

Eight pigs were assigned to the BA group and seven pigs to the non-BA group. In the non-BA group, apnea was observed in four of seven cases, three of which resulted in death. None of the eight pigs in the BA group had respiratory arrest; notably, all survived. Hypotension was observed in some pigs in each group; however, there were no cases of bradycardia in either group. Statistical analysis showed that wearing BA significantly reduced the occurrence of respiratory and cardiac arrest (*p*=0.026) but not survival (*p*=0.077). No significant differences were found in other vital signs.

Conclusions

Wearing BA with adequate neck and chest protection reduced mortality and it was effective to reduce cardiac and respiratory arrest against shock wave exposure. Mortality from shock wave injury appears to be associated with respiratory arrest, and the avoidance of respiratory arrest may lead to survival.

## Introduction

Ongoing fighting in the Gaza Strip in Palestine and the Russian invasion of Ukraine have resulted in blast injuries to soldiers and civilians. Japan has had few patients with blast injuries since World War II, and most have been cases of terrorist bombings and industrial explosions. However, given the current world situation, our country may face involvement in conflicts, battles, or invasions by other countries, making the need to respond to blast injuries a more imminent reality. Therefore, conducting basic research to prepare and save the lives of patients with blast injuries is necessary.

Blast injuries in modern society often occur due to terrorist attacks, particularly in urban settings, indoors, and in vehicles, leading to significant damage [1]. Therefore, it is important to focus on blast injuries in confined spaces rather than conventional open-field experiments.

In our previous study, we demonstrated the effectiveness of wearing bulletproof vests in saving lives [2]. The vest used in the experiment was a former type utilized by the defense force in our country, which was made of CORDURA® Ballistic Nylon and ceramic plate (Invista, Kennesaw, USA). Consequently, we established a significant correlation between respiratory arrest and mortality, suggesting that wearing a vest can prevent respiratory arrest. Therefore, based on the assumption that the survival rates increase when respiratory arrest is avoided, we designed a new steel body armor (BA) to protect the chest, which can trigger a nerve reflex for respiratory arrest, and the neck, which houses the medulla oblongata, the respiratory center, against shock waves to avoid immediate death due to blast injury. To the best of our knowledge, we have not been able to find any research on protective gear or BA that focuses on neck and chest protection.

The aim of this study was to show that respiratory arrest is highly associated with death in blast injuries, and adequate protection of the neck and chest increases the likelihood of survival from blast injuries under such conditions.

This article was previously presented as a poster at the Military Health System Research Symposium 2023 on August 16, 2023.

## Materials and methods

Animal preparation

**Figure 1:**
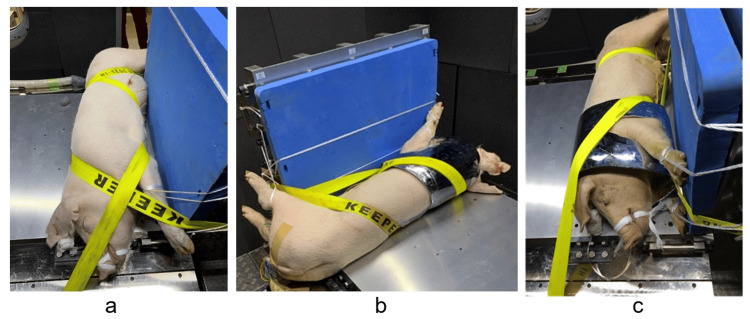
Pigs fixed on the experimental table in the measurement area a: a pig without body armor; b, c: a pig with body armor (b: viewed from dorsal side; c: viewed from oblique front side)

Male hybrid pigs (age, 10-12 weeks; mean body weight, 39.9 kg) were used in the experiments. Before the experiments, each animal was housed in an individual cage in a room with a 12:12 h light: dark cycle and an ambient temperature of 24℃. Animals were fed standard laboratory pig chow and had ad libitum access to water. The pigs were divided into two groups: a group not wearing a BA (non-BA group) and a group wearing a BA (BA group). The sample size was determined by taking into account that approximately half of the non-BA pigs were expected to die. Under anesthesia, tracheal intubation was performed, and a femoral arterial line was surgically secured to continuously monitor changes in vital signs. The electrodes of the electrocardiogram were implanted under the skin so they would not blow away. The pigs in the non-BA group were directly placed in the measurement area of the blast tube experiment room, while the pigs in the BA group were placed there after wearing the BA. The BA was shaped to protect the neck and chest and was made of steel. The pigs were fixed tightly in the right lateral position on the table in the measurement area so that the dorsal part of the pigs was positioned immediately in front of the aperture of the blast tube. Each limb was tightly fixed with a string, and their trunks were fixed to the table with two belts (Figure 1).

Anesthesia

**Figure 2:**
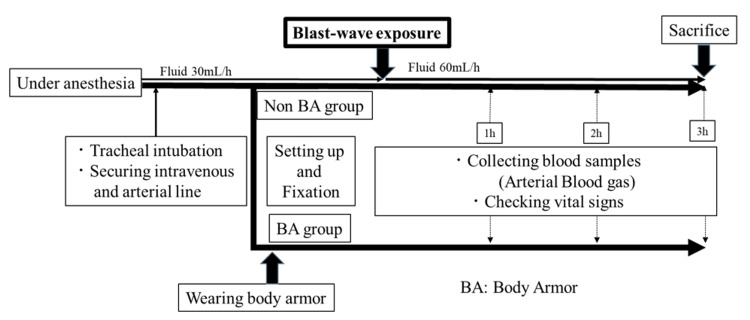
Study design of our experiment

All animals were anesthetized with medetomidine chloride (0.15 mg/kg) and midazolam (0.75 mg/kg) by subcutaneous injection. In the animal experimental room, we secured an intravenous line via the auricular vein and intravenously administered ketamine hydrochloride (25 mg/kg) and xylazine hydrochloride (15 mg/kg) every 30 min. Ringer's lactate solution was administered intravenously (30 mL/h) to maintain an intravenous line before the appliance of shock wave. If the pig was considered distressed, which was observed based on body movements, sequential additional doses of ketamine hydrochloride and xylazine hydrochloride were administered. After the shock wave exposure, Ringer's lactate solution was administered intravenously (60 mL/h) without fluid resuscitation. Surviving pigs were sacrificed after 3 hours. They were anesthetized with ketamine hydrochloride (150 mg/kg intravenous) and xylazine hydrochloride (90 mg/kg intravenous) for the procedure. All the protocol was prepared and approved before the study (Figure 2).

Blast tube setting

In 2017, we installed a blast tube, an air-driven shock wave generator, for basic research on blast injuries. It was installed indoors in a room 8 m long and 12 m wide. The tube itself was 8 m long and the opening aperture was 40 cm in diameter. The tube allows for evaluation using medium-sized animals as specimens, which can be considered comparable to humans. We conducted blast injury experiments using medium-sized pigs with a torso similar in size to the human body. Our institution is the only facility in Japan and among the few in the world for performing blast injury experiments on medium-sized animals indoors. We can set the driving pressure of our blast tube to 0.5, 1.0, 1.5, 3.0 and 5.5 MPa. In a previous experiment, we decided to conduct the experiment at a driving pressure of 3.0 MPa, so that the mortality rate would be approximately 50%, following the results of Bass et al. [3].

After the pig under anesthesia was fixed on the table and the measurement area was securely closed, we began to accumulate compressed air in the high-pressure and septal sections. The septal section is a small compartment next to the high-pressure section that stores approximately half of the pressurized air in the high-pressure section. After the high-pressure section reached 3.0 MPa, we instantly removed the compressed air from the septal section, which caused the septa to rupture. The released compressed air generated a shock wave, which we were able to measure.

Outcomes and measurement

We continuously observed changes in the vital signs before and after theappliance of the blast wave. Respiration was monitored and grossly observed. Dead specimens were dissected immediately after death, whereas live specimens were returned to the experimental room and observed for 3 hours after injury. Autopsies were performed to confirm the extent of organ damage. Dissection of the chest and abdomen, but not the brain, was performed. The amount of hemorrhage was checked and the organ damage was confirmed grossly. Comparisons were made between groups with respect to survival up to 3 hours after the exposure. Cardiac arrest, apnea, bradycardia, and hypotension right after the exposure, the last three of which are well-known as the triad of blast lung, were also compared. [4] Cases of incomplete rupture of blast tube septa were excluded as insufficient shock wave formation.

Statistical analysis

Statistical analysis was performed using Fisher's exact test, and statistical significance was set at *p*<0.05. Statistical analysis was performed using IBM SPSS Statistics ver. 29.0.1.0 for Windows (IBM Corp., Armonk, USA).

## Results

Samples

Fifteen male hybrid pigs were used in the experiments. Eight pigs were assigned to the BA group and seven pigs to the non-BA group.

Changes in vital signs

In the non-BA group, apnea was observed in four of the seven cases, three of which resulted in death. In contrast, none of the eight pigs in the BA group had respiratory arrest, and all survived. Hypotension appeared in four pigs in the BA group and in five pigs in the non-BA group; however, there were no cases of bradycardia in either group (Table 1).

**Table 1:** Results of vital sign changes in pigs with and without body armor after shock wave exposure

Armored	Apnea	Hypotension	Bradycardia	Arrest	Survive	Non-armored	Apnea	Hypotension	Bradycardia	Arrest	Survive
1	No	unknown	No	No	Yes	1	Yes	Yes	Unknown	Yes	Yes
2	No	No	No	No	Yes	2	Yes	Yes	Unknown	Yes	Yes
3	No	No	No	No	Yes	3	Yes	No	Unknown	Yes	Yes
4	No	Yes	No	No	Yes	4	No	unknown	No	No	No
5	No	Yes	No	No	Yes	5	No	Yes	No	Yes	No
6	No	unknown	No	No	Yes	6	No	Yes	No	No	No
7	No	Yes	No	No	Yes	7	Yes	Yes	No	No	No
8	No	Yes	No	No	Yes						

Statistical analysis

Statistical analysis showed that wearing BA significantly reduced respiratory and cardiac arrest (*p*=0.026) but not survival (*p*=0.077). No significant differences were found in other vital signs.

Autopsy

Autopsy results showed bilateral pulmonary contusions and intra-abdominal hemorrhage due to splenic injury in most cases (Table 2).

**Table 2:** Organ injuries after shock wave exposure LC: Lung Contusion; IAH: Intraabdominal hemorrhage; SI: Spleen injury Hemorrhage: slight: ~100ml, mild: 100~300ml, moderate: 300~600ml

armored	Remarks on organ damage(by sacrifice)	Non-armored	Remarks on organ damage(by sacrifice)
1	Bilateral LC, slight IAH due to SI	1	Bilateral LC, moderate IAH due to SI, Bladder rupture
2	Bilateral LC, moderate IAH due to SI	2	Bilateral LC
3	Bilateral LC, moderate IAH due to SI	3	Bilateral LC, mild IAH due to SI, multiple rib fracture
4	Bilateral LC, lt peumothorax, rib fracture, slight IAH due to SI	4	Bilateral LC, mild IAH due to SI
5	Bilateral LC, slight IAH due to SI	5	slight bilateral LC, moderate IAH die to SI
6	Bilateral LC	6	Bilateral LC, moderate IAH due to SI
7	Bilateral LC, moderate IAH due to SI	7	Bilateral LC, moderate IAH due to SI
8	None		

## Discussion

In the BA group, not a single case of respiratory arrest was observed, and all pigs survived. In the non-BA group, respiratory arrest occurred in four of the seven pigs, and three of them died. All of the deaths occurred after respiratory arrest. No cases of bradycardia were observed in either group and therefore, none of the pigs in both groups fulfilled the triad of blast lung. All but three pigs had pulmonary contusions and splenic injuries. Most of the cases exhibited only moderate hemorrhage, which was associated with splenic injury. Thus, hemorrhage was not considered associated with death. Notably, one of the pigs in the non-BA group had no intra-abdominal hemorrhage, yet it died, suggesting respiratory arrest as the cause of death.

To save lives against blast injuries, it is extremely important to clarify which areas of the body should be protected, taking into account the physiological reactions of the body immediately after exposure to the blast wave and the kind of biological reactions it may induce. One of the most well-known physiological responses to blast exposure in the thorax is the triad of blast lung, consisting of apnea, bradycardia, and hypotension [4]. Bradycardia and apnea are both considered mediated by a vagal reflex [5-8], with the most likely candidate being the pulmonary afferent C-fiber reflex [9]. Hypotension may be due to a complex response other than the neural reflex, such as vasodilation caused by nitric oxide release and other factors [10]. However, in reality, the whole body is instantly exposed to the blast wave; therefore, not only the pulmonary nerve reflexes are remarkable. In most ideal models where the shock wave is applied only to the thorax, these three signs can be seen. However, when the shock wave is applied to the whole body, as in this study, the triad of the blast lung is not always clear. In this study, there was not a single case of bradycardia, let alone a case that met the triad of blast lung. In our previous study, respiratory arrest was considered likely associated with mortality [2], and all deaths were associated with respiratory arrest, suggesting a relationship between mortality and respiratory arrest.

In addition to protecting the chest against a shock wave, we considered it important to protect the neck, where the medulla oblongata resides, to avoid direct impact or stimulation of the medulla oblongata where the respiratory center itself is located. Therefore, based on the hypothesis that it would be possible to save lives from blast injury if both the neck and chest were protected, we applied the BA on pigs before conducting experiments. In this study, BA probably prevented respiratory and cardiac arrest, but not mortality. Further consideration should be given to which area should be prioritized for further protection against blast injury: the cervical (medulla oblongata) or the thoracic area. We used a device that generated localized shock waves with a laser (laser-induced shock waves [LISW]) and applied LISWs to the frontal and posterior neck, as well as chest regions of mice, and showed that the mortality rate was significantly higher in the group mice exposed to LISW in the posterior neck region [11]. These results indicate that when a living body is exposed to a shock wave, its effect on the neck contributes the most to life expectancy. Therefore, the use of protective equipment to enhance neck protection is important. Previous studies have reported that BA is not always effective against blast injuries to save lives. Phillips et al. reported that wearing an army ballistic jacket did not protect against shock wave damage to the chest in basic blast injury experiments using sheep [12]. In a report on direct human blast injuries, Mellor et al. stated that 828 servicemen were killed and injured by explosions in Northern Ireland, and 90% of them wore BA, leading to the conclusion that BA can provide considerable protection against secondary missiles, but will not reduce the number of deaths from primary blast injuries [13]. However, these BA or ballistic jackets might not have been sufficient for neck protection. Therefore, to save the lives of those injured by blast waves, especially in the super-acute phase, it may be necessary to fabricate protective devices that provide greater protection to the neck area to protect the medulla oblongata.

Our findings indicated that BA protecting the neck and chest may avoid respiratory arrest, and although the small number of samples did not allow us to show significant differences, we believe that BA may improve survival against shock waves.

In the future, we plan to develop a new BA with enhanced neck protection and examine its effectiveness. We also plan to administer respiration-stimulating agents to avoid respiratory arrest, as well as examine the possibility of increasing survival rates against shock wave exposure.

This study has some limitations. First, the sample size was small and insufficient to draw exact conclusions. Second, although the pigs were sufficiently fixed in this study, their bodies were blown afloat, which introduced the effects of tertiary blast injuries in addition to those of primary blast injuries caused by the shock wave. Third, continuous monitoring under the effect of significant blast shock waves was challenging, particularly in the early stages. Multiple adjustments and modifications were necessary. The monitoring cables and measuring instruments were sometimes disconnected or damaged, resulting in missing data. Finally, as the shock wave travels through the blast tube and diffuses at the aperture, it could have a different effect on the body than an actual blast with a spherical shock wave.

## Conclusions

Wearing BA with adequate neck and chest protection reduced mortality and was effective in reducing cardiac and respiratory arrest against shock wave exposure. Mortality from shock wave injury appeared to be associated with respiratory arrest; therefore, the avoidance of respiratory arrest may lead to survival.
